# Expression levels and polymorphisms of the microRNA maturing components; diagnostic values of Drosha, DGCR8 and Dicer in patients with vitiligo

**DOI:** 10.4314/ahs.v25i2.19

**Published:** 2025-06

**Authors:** Soner Aşır, Özlem İzci Ay, Mustafa Ertan Ay, Kenan Çevik, Gurbet Doğru Özdemir, Merve Türkegün Şengül, Mehmet Emin Erdal, Ümit Türsen

**Affiliations:** 1 Mersin University, Faculty of Medicine, Department of Medical Biology and Genetics; 2 Alanya Alaaddin Keykubat University, Faculty of Medicine, Department of Biostatistics and Medical Informatics; 3 Mersin University, Faculty of Medicine, Department of Dermatology

**Keywords:** Vitiligo, miRNA biogenesis, Dicer, Drosha, DGCR8

## Abstract

**Background and Objective:**

Even though the pathogenesis of vitiligo is still unclear, recent studies have suggested that miRNAs can contribute to the occurrence and progression of the disease. The aim of the present study was to investigate the associations between SNPs of miRNA processing genes and their expression levels with vitiligo susceptibility.

**Methods:**

55 patients and 56 controls were investigated for Dicer, Drosha, and DGCR8 gene expressions and genotyped for Drosha rs493760, DGCR8 rs1640299, and Dicer rs1057035 by real-time PCR. The correlation of the expression levels of these three genes was analyzed. The ROC curve was used to analyze their diagnostic efficacy for vitiligo.

**Results:**

The current findings showed that the Dicer CT genotype was more frequent in vitiligo (p=0.046) compared to controls, while Drosha and DGCR8 polymorphisms did not show significant associations. The relative expression levels of the three genes in vitiligo patients were significantly lower than those in the control group (p<0.05). The areas under the curves for Drosha, DGCR8, and Dicer were 0.969, 0.66, 0.67.

**Conclusions:**

For the first time, we demonstrate that the Dicer rs1057035 polymorphism is associated with vitiligo susceptibility, and the downregulation of Drosha, DGCR8, and Dicer suggests their potential roles as biomarkers in the pathogenesis of vitiligo.

## Introduction

Vitiligo is the most common depigmentary skin disorder, characterized by the appearance of white patches on the skin, resulting from the selective destruction of melanocytes. Accumulating evidence reports that vitiligo can affect the general population with a frequency varying between 0.1% and 2% across different geographic distributions and ethnic groups^1^. Both genders are equally affected. Although it mostly affects young adults, it can develop at any age^2^,^3^. Segmental (SV) and non-segmental vitiligo (NSV) are the two main types of the condition, based on clinical grounds. NSV, the most prevalent type of vitiligo, is characterized by symmetrical, bilateral white spots, whereas SV often shows a unilateral distribution throughout the body^4^,^5^. The mechanism underlying vitiligo has not been fully elucidated, but various theories, including the autoimmune hypothesis, genetic theory, and biochemical, molecular, and cellular defects, have been proposed to explain its pathogenesis^6^,^7^. A great number of reports suggest that multiple factors contribute to the occurrence and development of vitiligo and that it is polygenic. Noncoding RNAs may have a crucial role in vitiligo susceptibility. Recent studies have focused on miRNAs in the emergence of vitiligo.^8^ miRNAs are small non-coding RNAs about 22 nucleotides in length^9^. miRNA biosynthesis begins with the transcription of miRNA genes into primary miRNA (pri-miRNA) transcripts via RNA polymerase II or RNA polymerase III. The hairpins found in pri-miRNA are recognized by a nuclear protein known as the DiGeorge syndrome critical region, which is associated with the Drosha protein. The Drosha-DGCR8 complex, also known as the microprocessor complex, releases the hairpins from pri-miRNAs by cleaving approximately eleven nucleotides from the hairpin base to produce pre-miRNAs. Next, the pre-miRNA containing 70-90 nucleotides is transported into the cytoplasm via exportin-5. In the cytoplasm, Dicer, with RNase III enzyme activity, processes the pre-miRNA to form mature miRNA with the desired function. Thus, it seems that the roles of Drosha, Dicer, and DGCR8 as major components of the miRNA biogenesis pathway are inevitable^10^,^11^.

As highly conserved, noncoding small RNAs, miRNAs have important effects on various physiological and developmental processes in humans. Recently, many studies have indicated that they participate in diverse essential aspects of the pathological process of vitiligo, including the immune response, apoptosis, and the differentiation and growth of melanocytes^12^,^13^. Numerous reports have demonstrated aberrant expressions of miRNAs in the skin, serum, and peripheral blood mononuclear cells of vitiligo patients. For example, miR-16, miR-19b, and miR-720 are effective serological markers that differentiate NSV from healthy individuals^14^.

Given that miRNA dysregulation is linked to vitiligo and that miRNA expression levels can be influenced by SNPs and changes in the expression of miRNA machinery genes, we hypothesized that vitiligo may be associated with miRNA machinery gene polymorphisms and expressions. Therefore, we aimed to analyze the association between the expression levels of miRNA machinery genes (Drosha, DGCR8, Dicer) and SNPs (Drosha rs493760, DGCR8 rs1640299, Dicer rs1057035) in three key components of miRNA machinery genes and the risk of vitiligo. The selected SNPs are located in the intronic, 3′UTR, and 5′UTR regions, respectively. Intronic polymorphisms were chosen because they can affect gene expression through their effects on splicing, regulatory elements, mRNA stability, noncoding RNAs, and chromatin structure. Polymorphisms in the 3′ UTR and 5′ UTR were chosen because they can have significant effects on the expression levels and functional output of a gene by affecting various regulatory mechanisms (binding efficiency of transcription factors, mRNA stability, changes in binding efficiency of non-coding RNAs, changes in mRNA stability and degradation rates, etc.) at both transcriptional and post-transcriptional levels. We believe that it is important to investigate these Drosha, DGCR8, and Dicer SNPs and expressions for the first time in vitiligo.

## Materials and Methods

### Study subjects

The study group included 55 volunteer patients diagnosed with vitiligo in the Dermatology Department of Mersin University Research Hospital. The diagnosis of vitiligo was confirmed clinically. Clinical and demographic features, including age and gender, were recorded. Patients who were not diagnosed with non-segmental vitiligo in the dermatological examination and controls who were diagnosed with any autoimmune disease or had a family history of vitiligo were excluded from the study. As a control group, fifty-six healthy individuals with no other autoimmune diseases, skin disorders, or family history of vitiligo were selected and matched with the patient group in terms of age and sex. Written informed consent was obtained from all participants in the present study, and ethical approval was acquired from the Ethics Committee of Mersin University Clinical Research (Approval number 2019/380).

### Selected Genes and SNPs

Table 1 demonstrates the name, SNP ID, chromosomal location, polymorphisms, position, and nucleotide changes of all three SNPs and genes that are key regulators for mature and functional miRNAs. The selected SNPs have a reported minor allele frequency of >0.01 in whites. Drosha (rs493760) is located in introns, while Dicer (rs1057035) and DGCR8 (rs1640299) are located in the 3′UTR regions of the genes.

**Table 1:** SNPs identified in *Drosha, DGCR8* and *Dicer* genes

Gene	*Gene ID*	Chr *location*	*SNP rs no*	*Position*	*Variant (M>m)*
*Drosha*	29102	chr5:31436933	rs493760	intron	C>T
*DGCR8*	54487	chr22:20098359	rs1640299	3′-UTR	G>T
*Dicer*	23405	*chr*14: 95087805	rs1057035	3′-UTR	T>C

Chr, chromosome; dbSNP, SNP location on reference genome in SNP data bank; M, major allele; m, minor allele; SNPs, singlenucleotide polymorphisms; UTR, untranslated region

### Genotyping

The study group included 55 volunteer patients diagnosed with vitiligo. Blood samples were collected from all participants into tubes containing 2% EDTA (Sigma E-5134, Germany) to isolate the genomic DNA. All samples were transferred to our molecular analysis laboratory, where DNA isolation was performed using the Hibrigen DNA isolation kit (MG-GDNA-01). The quality and quantity of each DNA sample were measured with a Nanodrop® spectrophotometer (Thermo Scientific, Wilmington, DE, USA). DNA samples were genotyped for Drosha (rs493760), DGCR8 (rs1640299), and Dicer (rs1057035) genes using the TaqMan SNP genotyping assay in the 7500 Real-Time PCR System with a 96-well plate (Applied Biosystems, Foster City, CA, USA).

The final real-time PCR was carried out in a 20 µL reaction volume containing 30 ng DNA, 12.5 µL 2X TaqMan Universal PCR Master Mix, 900 nmol primers, 200 nmol probes, and nuclease-free water (Applied Biosystems, Foster City, CA, USA). The reaction conditions included 2 minutes of pre-incubation at 50°C, enzyme activation at 95°C for 10 minutes, followed by 40 cycles of denaturation at 95°C for 15 seconds, and 40 cycles of annealing and extension at 60°C for 1 minute. Table 2 shows the primers and probes designed using Primer Express 3.0 software (Applied Biosystems, Foster City, CA). All reactions were performed in triplicate.

**Table 2:** Primer-probe sequences of *Drosha, DGCR8* and *Dicer* genes for genotyping

Genes	Primer and Probe Sequences
*Drosha* (rs493760)	F: 5′-AAAGACAAATCCTAGAAGATGAAATGACA-3′ R: 5′-R-AGATCAGCTTGCCTTGGTCTAGA-3′ PR: 5′-Yakima Yellow-CTTTACACACGCGCT**C**AGGGCA-BHQ-1-3′ PR: 5′-FAM-CTTTACACACGTGCTCAGGGCAACC-BHQ-1-3′
*DGCR8* (rs1640299)	F: 5′-TGGCCTCCTAGGGTCCCTT-3′ R: 5′-AAGGCAGAGAGGGCCTCAGT-3′ PR: 5′-Yakima Yellow-T(pdC)TTAAT**T**C(pdC)CTAAAAG(pdC)GCCT-BHQ-1-3′ PR: 5′-FAM-TCTTAAT**G**C(pdC)CTAAAAG(pdC)GCCT-BHQ-1-3′
*Dicer* (rs1057035)	F: 5′-TCTGCAGTTGCTTTTTCAAGACA-3′ R: 5′-GAGACCGAATGTAATATGGAAAACCT-3′ PR: 5′-Yakima Yellow-CTTTACACACGCGCT**C**AGGGCA-BHQ-1-3′ PR: 5′-FAM-CTTTACACACGTGCTCAGGGCAACC-BHQ-1-3′

BHQ:Black Hole Quencher; F:forward; R:reverse; PR:probe. Bold nucleotides in the probe sequences indicate SNPs and the substitution of C-5 with dC, which is an effective strategy for enhancing base pairing (pdC: propynyl-dC)

### Total RNA Isolation and cDNA Synthesis

Total RNA from all blood samples was extracted using TRIzol reagent (Invitrogen). Each RNA sample was quantified at 260 nm (40 ng/ml OD) using a spectrophotometer (Thermo Scientific, Wilmington, DE, USA), and the purity of RNA samples was assessed by the ratio of readings at 260 nm and 280 nm. Using gene-specific primers as shown in Table 2, cDNAs were synthesized from total RNA. Reverse transcriptase PCR reactions for the three genes included 2 µg of extracted total RNA, 200 U of Revertaid Reverse Transcriptase (Thermo Scientific, Vilnius, Lithuania), 5× RT buffer, poly-T primer, 2 mM each of dNTPs, 40 U of RiboLock RNase inhibitor (Thermo Scientific, Vilnius, Lithuania), and nuclease-free water to a final reaction volume of 50 µL. The 50 µL reactions were incubated for 60 minutes at 37°C, 5 minutes at 95°C, and then held at 4°C. Real-time PCR was performed using a Bio-Rad MiniOpticon Real-time PCR Detection System (Bio-Rad, CA). cDNAs were stored at −20°C until Real-time PCRanalysis.

### Quantitative Real-time PCR (qPCR) Analysis

The expression of Drosha, DGCR8, Dicer, and ACTB transcripts were measured by ABI Prism 7500 Real-Time PCR System (Applied Biosystems) using the SDS 2.0.6 software and specific primers/probes for selected genes as shown in Table 3. The expression of the ACTB gene was used as a reference. The 25 µL real-time PCR reactions were performed in duplicates and included 12.5 µL of TaqMan Gene Expression Master Mix (Applied Biosystems), 5 µL of cDNA, primers (1 µL each), probes (0.6 µL each), and nuclease-free water. The reaction conditions included a pre-incubation at 50°C for 2 minutes and denaturation at 95°C for 10 minutes, followed by 50 cycles of 95°C for 15 seconds and 60°C for 1 minute. The real-time growth curves were analyzed in the logarithmic chart in SDS 2.0.6, and the expression levels were determined using the 2-ΔΔCT method.

**Table 3:** Primer-probe sequences of selected genes used in Real-time PCR for gene expressions

Genes	Gene ID	Primer-probe sequences
*Dicer*	23405	F-5′-CCCGGCTGAGAGAACTTACG-3′
		R-5′-TGTAACTTCGACCAACACCTTTAAAT-3′
		PR-5′-FAM-CGGGAAGGT(pdC)AGAGT(pdC)A-ZNA4 BHQ-1-3′
*Drosha*	29102	F-5′-GAACAGTTCAACCCCGATGTG-3′
		R-5′-CTCAACTGTGCAGGGCGTATC-3′
		PR-5′-FAM-TTA(pdC)TTTT(pdC)CGATTAT(pdC)GTC-ZNA4-BHQ-1-3′
*DGCR8*	54487	F 5′-TCTTTGAATGTGAGAACCCAAGTG-3′
		R 5′-CCGTAAGTCACACCATCAATGG-3′
		PR 5′-FAM-CCTTTTGGTGCCTCGGT-ZNA4-BHQ-1-3′
*ACTB*	60	F 5′-GGCACCCAGCACAATGAAG-3′
		R 5′-GCCGATCCACACGGAGTACT-3′
		PR 5′-Yakima Yellow-TCAAGATCATTGCTCCTCCTGAGCGC-BHQ-1-3′

### Statictical Analysis

MedCalc demo version 20.115 was used for statistical analysis of the data. Normal distribution control for numerical variables was performed with the Shapiro-Wilk test. Numerical variables that did not show a normal distribution were summarized by the median value and interquartile range (IQR). The distribution of categorical variables was summarized with numbers and percentages. A chi-square test was used for Hardy-Weinberg equilibrium control in the patient and control groups, and it was interpreted that HW equilibrium was ensured for p>0.05. The medians of age and gene expression levels were compared between the patient and control groups with the Mann-Whitney U test, and Box and Whisker plots were plotted. The medians of gene expression levels were compared between genotype groups with the Kruskal-Wallis test. Relationships between genotype and groups were analyzed by chi-square analysis. Spearman's rho correlation coefficient was used to investigate the relationships between gene expression levels, and scatter plots were drawn. ROC analysis was performed to evaluate the diagnostic performance of expression levels in patient-control groups, and ROC curves were drawn. Sensitivity, specificity, and AUC (Receiver Operating Characteristics Curve) statistics and their 95% confidence intervals were calculated, and those with a confidence interval that did not include the “1” value were considered statistically significant. A statistical significance level (p) ≤0.05 was taken for all comparisons.

## Results

The present case-control study enrolled 55 Turkish patients with vitiligo. Among them, 27 (49.1%) were males and 28 (50.9%) were females, with a median age of 23 years. The control subjects included 29 (51.8%) males and 27 (48.2%) females, with a median age of 28.5 years. No significant difference was observed in terms of age and sex between the vitiligo patients and the control group (p=0.761 and p=0.776, respectively).

### Drosha, DGCR8 and Dicer polymorphisms

Allele frequencies for Drosha (rs493760 C/T), DGCR8 (rs1640299 G/T) and Dicer (rs1057035 T/C) are given in Table 4, and genotype frequencies are given in Table 5. Drosha, DGCR8, and Dicer in the vitiligo patient group were in Hardy-Weinberg equilibrium (p>0.05). In the control group, only Dicer was not in Hardy–Weinberg equilibrium (p<0.05) (Table 4).

**Table 4:** Allele frequencies for Drosha (rs493760 C/T), DGCR8 (rs1640299 G/T) and Dicer (rs1057035 T/C) in patient and control groups

Patient					Control				
Hardy-Weinberg Equilibrium		n	Allel frequency	p value for *χ*^2^	*χ* ^2^	n	Allel frequency	p value for *χ*^2^	*χ* ^2^
*Drosha*	C T	24 78	0.24 0.76	0.521	0.41 0	24 84	0.22 0.78	0.066	3.375
*DGCR8*	G T	61 45	0.58 0.42	0.756	0.09	55 55	0.50 0.50	0.892	0.018
*Dicer*	T C	21 87	0.19 0.81	0.365	0.81 8	14 94	0.13 0.87	**0.01**	6.37

As shown in Table 5 in Drosha (rs493760 C/T) and DGCR8 (rs1640299 G/T), there were no differences in the genotype and allele frequencies of these polymorphisms between the patients and controls. In Dicer (rs1057035 T/C), CT genotypes were significantly more frequent in patient group than that in control group (p=0.046).

**Table 5:** Genotype Distributions of Drosha (rs493760 C/T), DGCR8 (rs1640299 G/T) and Dicer (rs1057035 T/C) SNPs in Patients and Controls

Variables	Patients (n=55)	Control (n=56)	x^2^	p-value
***Drosha rs493760***				

CC, n (%)	2 (3.9)	5 (9.3)	2.824	0.257
CT, n (%)	20 (39.2)	14 (25.9)		
TT, n (%)	29 (56.9)	35 (64.8)		
HWE-p	0.9	0.6		

***DGCR8 rs1640299***				

GG, n (%)	17 (32.1)		1.341	0.532
GT, n (%)	27 (50.9)			
TT, n (%)	9 (17.0)			
HWE-p	0.08	0.4		

***Dicer rs1057035***				

CC	34 (63.0)	43 (79.6)	6.533	**0.046**
CT	19 (35.2)	8 (14.8)		
TT	1 (1.9)	3 (5.6)		
HWE-p	0.2	0.7		

*HWE-p: Hardy-Weinberg equilibrium p value. p values<0.05 are indicated in bold*.

**Fig 1:**
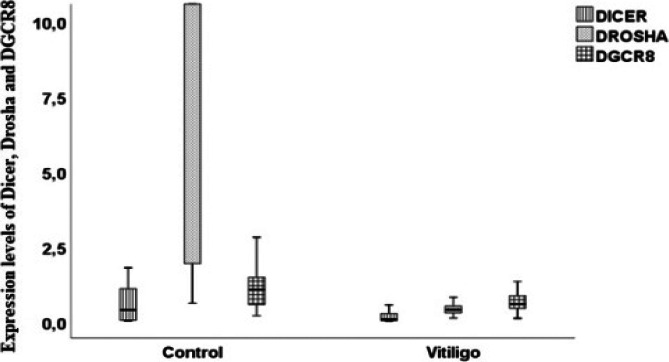
The relative expression levels of Drosha, DGCR8 and Dicer in vitiligo patients compared with the control subjects

Drosha, DGCR8 and Dicer gene expression levels and the genotypes of each polymorphism Fig 1. The relative expression levels of Drosha, DGCR8 and Dicer in vitiligo patients compared with the control subjects.

Drosha, DGCR8 and Dicer gene expressions showed no significant difference among any genotypes of Drosha, DGCR8 and Dicer polymorphisms (p>0.05) (Table 6).

**Table 6:** Correlations between *Drosha*, DGCR8 and *Dicer* expressions and polymorphisms in vitiligo and controls

Genotype		n	Patient Median (IQR)	pa	Control Median (IQR)	n	pb
Dicer Expression							
Dicer	CC	34	0.070 (0.241)	0.584	0.357 (0,969)	43	0.672
	TT	1	0.023 (0.000)		1.170 (---)	3	
	CT	19	0.044 (0.301)		0.494 (1.220)	8	
Drosha Expression							
Drosha	TT	34	0.385 (0,166)	0.448	10.832 (17.157)	43	0.511
	CC	1	0.285 (---)		8.852 (24.759)	3	
	TC	19	0.332(0,254)		14.791 (24.598)	8	
DGCR8 Expression							
DGCR8	GG	14	0.658 (0.443)	0.051	1.082 (1.136)	14	0.816
	TT	14	0.496 (0.320)		0.846 (0.933)	14	
	GT	27	0.472(0.438)		1.071 (0.941)	27	

*IQR: Interquartile range; (---): IQR could not be calculated due to insufficient observation values. pa: p value of Kruskal- Wallis test at patient groups, pb: p value of Kruskal Wallis test at control groups*.

### The diagnostic values of Drosha, DGCR8 and Dicer genes in vitiligo

**Fig 2:**
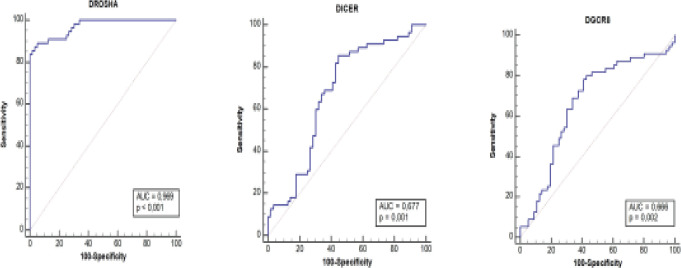
ROC curve analysis for the expression of Drosha, DGCR8 and Dicer. The area under the curve (AUC) values indicate 2-AACT. According to the results, Drosha has excellent diagnostic values forifferantiating the patients from the controls, DGCR8 and Dicer have acceptable values

Since the expression levels of all three genes differed significantly between patient and control groups, the diagnostic performance of expression values in patients with vitiligo were evaluated. The findings demonstrated that Drosha, DGCR8 and Dicer genes have significant sensitivity and specificity (p < 0.05). As shown in Fig 2, ROC curve analysis revealed that the AUC area of DGCR8 for vitiligo diagnosis was 0.666, the sensitivity of 80%, and the specificity of 57.14% [95% confidence interval (CI), 0,57-0,75; p = 0.002]; the AUC area of Dicer was 0.677, the sensitivity of 85.45%, and the specificity of 55.36% [95% CI, 0,582-0,763; p = 0.001]; the AUC area of Drosha was 0.969, the sensitivity of 89.09%, and the specificity of 94.64% [95% CI, 0,918-0,993; p < 0.001]. These results suggest that Drosha has strong diagnostic value in differentiating healthy individuals from patients with vitiligo.

### Correlation analysis of the relative expressions of Drosha, DGCR8 and Dicer genes

**Fig 3:**
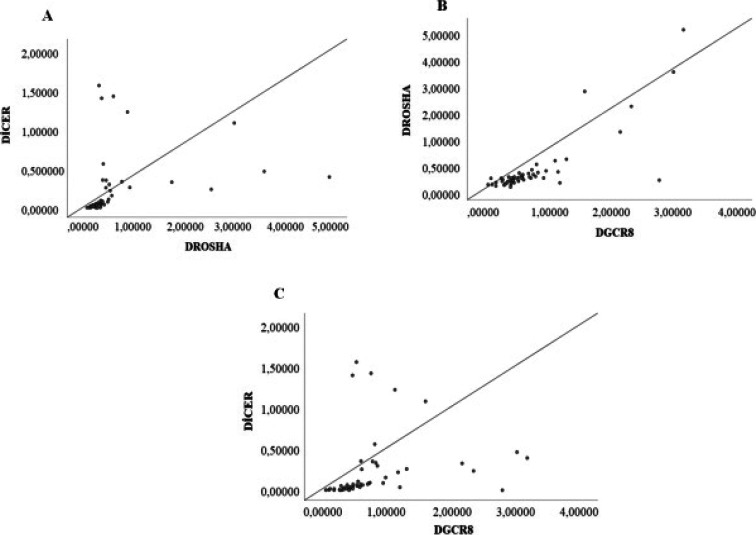
The correlation analysis between three genes (A) correlation between Dicer and Drosha; (B) correlation between Drosha and DGCR8; (C) correlation between Dicer and DGCR8

Correlation analysis of the relative expression levels of Drosha, DGCR8 and Dicer genes in the total 111 research objects from vitiligo and control groups was performed using Spearman's rank correlation analysis. A weak positive correlation between Dicer and Drosha (r=0.303, p=0.01), Drosha and DGCR8 (r=0.388, p<0.001) and a moderate positive correlation between Dicer and DGCR8 (r=0.633, p<0.001) was found. In patient group, the expression levels between Dicer and Drosha (r=0.833, p<0.001) showed very high positive correlation. Dicer and DGCR8 (r=0.721, p<0.001) and Drosha and DGCR8 genes (r=0.771, p<0.001) showed high correlations (Fig 3).

## Discussion

In 2009, Yi and colleagues reported that both Dicer and DGCR8 play crucial roles in the control of mammalian skin development due to their function in regulating miRNA biogenesis, based on studies conducted on Dicer and DGCR8 Skin Conditional Knockout Animals^15^. In recent years, the identification of miRNAs in dermatological diseases has become a rapidly growing area of research, although the number of studies conducted remains limited. Dysregulation of miRNAs has significant effects on the pathogenesis of various human diseases, including skin disorders. As critical regulators of gene expression, miRNAs control epidermal differentiation, proliferation, and inflammation. Thus, it is not surprising that they are important in vitiligo as well as other skin diseases. The impact of miRNAs in skin diseases or development has been highlighted in various studies using animal models, targeting key regulators of miRNA machinery including Drosha, DGCR8, and Dicer^16^,^17^. Due to their essential roles in miRNA maturation, we focused on the polymorphisms and gene expression profiles of Dicer, Drosha, and DGCR8, which act upstream in the miRNA biogenesis pathway, in patients with vitiligo compared to healthy controls. To our knowledge, no studies have assessed these miRNA machinery genes—Drosha, DGCR8, and Dicer—in patients with vitiligo, making this the first report highlighting the importance of SNPs and expression levels of these key regulators in vitiligo pathogenesis.

Drosha, DGCR8, and Dicer are important in common diseases. Dicer depletion can lead to severe developmental abnormalities in many organs and embryonic lethality. A recent study showed that reduced Dicer expression in melanocytes, both in mouse genetic models and human and mouse cell lines, results in cell misplacement within hair follicles. This misplacement impairs melanin transfer to keratinocytes in growing hair and depletes the melanocyte stem cell pool^18^. Changes in miRNA biogenesis gene expression have also been associated with diabetic skin^19^ and epithelial skin cancers^20^. A study on type 1 and type 2 diabetic mouse models emphasized that reduced expression of DROSHA, DGCR8, XPO5, DICER1, and AGO2 leads to a general downregulation of miRNAs in diabetic skin^19^. Dysregulation in miRNA biogenesis genes and consequent changes in miRNA levels and functions may contribute to epithelial skin carcinogenesis^20^. Keratinocyte-specific deletion of Dicer and DGCR8 resulted in dramatic skin phenotypes due to a lack of mature miRNAs, indicating that genetic defects in miRNA expression may lead to skin diseases^17^. For example, miR-203 was the first miRNA studied in the skin. Its location at the transition between the dividing and differentiating layers of the epidermis suggested that the hyperproliferation phenotype observed in Dicer knockout skin may be due to the loss of miR-203^21^.

Our results showed that Drosha, DGCR8, and Dicer were significantly downregulated in vitiligo patients compared to controls. Ye et al. observed that the mTOR-Mdm2-Drosha pathway is involved in cellular responses to nutrient and energy deprivation^22^. Growth factors may protect against oxidative stress-induced apoptosis by activating the AKT and mTOR pathways. Indirect evidence suggests that α-melanocyte-stimulating hormone (MSH) stimulates melanogenesis through activation of MEK/extracellular signal-regulated kinase (ERK) or PI3K/AKT^23^. Modulation of the PI3K/AKT/mTOR signaling pathway could offer a novel approach for managing vitiligo according to our study.

Vitiligo is also known as an autoimmune disease. Low expressions of Drosha, DGCR8, and Dicer may reduce certain miRNAs, such as miR-27b, miR-let7f, miR-21, and miR-98, which are associated with the immune system or autoimmune diseases. Additionally, Dicer-knockout mice develop uncontrollable autoimmune diseases^24^. Therefore, downregulated expressions of Drosha, DGCR8, and Dicer in vitiligo patients may decrease such miRNAs and increase susceptibility to vitiligo. ROC analysis showed that low expressions of Drosha, DGCR8, and Dicer could distinguish vitiligo cases from healthy controls, suggesting that these genes may be potential biomarkers for accurate diagnosis of vitiligo.

In our study, no significant statistical differences were observed between vitiligo patients and controls regarding the frequencies of Drosha and DGCR8 gene polymorphisms in the Turkish population. However, for the Dicer polymorphism, the CT genotype was more frequent in vitiligo patients compared to controls, indicating that the Dicer CT genotype may be significantly associated with an increased risk of vitiligo. This polymorphism (rs1057035 C>T) is located in the 3′-UTR region of the Dicer gene. Several reports suggest that this variant may regulate Dicer expression by interfering with the binding of hsa-miR-574-3p. Although its exact function is unclear, this miRNA targets several hundred human genes. It is speculated that the C allele (not the T allele) might affect the binding of hsa-miR-574-3p, leading to decreased expression of the Dicer gene and thus increasing the risk of vitiligo^25^,^26^.

Genetic variants in the DGCR8 gene have been associated with schizophrenia, primary open-angle glaucoma, and various cancers, including laryngeal and bladder cancers, but their impact on vitiligo has not been elucidated^27^. DGCR8 polymorphism (rs1640299 G>T) is located in the 3′-UTR region of the gene and may affect its expression levels^28^. Our results suggest that this polymorphism may not be a genetic risk factor for vitiligo development. Drosha polymorphism (rs493760) is located in intronic region of this gene and very few studies with this gene polymorphism have been conducted on according to literature^29^-^31^. We found no relationship between this polymorphism and vitiligo. Additionally, no differences were observed in the expressions of Drosha, DGCR8, and Dicer genes among the genotypes of each polymorphism. The fact that only one polymorphism per gene was investigated suggests that other polymorphisms may also be relevant. Therefore, further examination of other polymorphisms in genes involved in the miRNA formation pathway is important for understanding vitiligo pathogenesis.

A comprehensive transcriptomic study published in 2024 found that in vitiligo patients, the expression of genes regulating melanogenesis, cell adhesion, cell survival, proliferation, and development is restructured compared to perilesional areas. We believe that functional changes in miRNA biogenesis genes, which are the focus of our research, may lead to changes in miRNA levels, altering the expression of many genes and potentially contributing to the etiopathology of vitiligo and other diseases^32^.

Our study has several strengths and limitations. First, to our knowledge, this is the first study to examine the association between three different SNPs (Drosha rs493760 C>T, DGCR8 rs1640299 G>T, and Dicer rs1057035 T>C) in miRNA machinery genes and vitiligo risk in the Turkish population, finding a positive association between rs1057035 in the 3′-UTR of Dicer and increased vitiligo risk. Second, the relationships of these SNPs with vitiligo in other ethnic groups require further validation through larger prospective studies. Additionally, the mechanisms by which the Dicer rs1057035 polymorphism increases vitiligo risk are still unknown, warranting further characterization in vitro and in vivo. Lastly, our study group may be relatively small.

In conclusion, our study provides the first evidence that downregulated expression levels of Drosha, DGCR8, and Dicer may be involved in vitiligo pathogenesis, and that the Dicer rs1057035 polymorphism may contribute to increased susceptibility to vitiligo in the Turkish population. Well-designed, population-based studies with larger sample sizes and diverse ethnic groups are needed to confirm and extend our findings.

## Data Availability

Data will be made available on reasonable request.
